# To Bees or Not to Bees: Greater Honeyguides Sometimes Guide Humans to Animals Other Than Bees, but Likely Not as Punishment

**DOI:** 10.1002/ece3.71136

**Published:** 2025-04-28

**Authors:** David J. Lloyd‐Jones, Musaji Muamedi, Claire N. Spottiswoode

**Affiliations:** ^1^ FitzPatrick Institute of African Ornithology, Department of Biological Sciences University of Cape Town South Africa; ^2^ Mbamba Village, Niassa Special Reserve, Niassa Province Mozambique; ^3^ Department of Zoology University of Cambridge Cambridge UK

**Keywords:** greater honeyguides, *Indicator indicator*, indigenous ecological knowledge, mutualism, punishment, spatial error

## Abstract

Greater honeyguides ( *Indicator indicator* ) are well known to guide human honey hunters to wild bees' nests in exchange for beeswax as food. Centuries of African Indigenous accounts have intriguingly reported that honeyguides occasionally guide humans to animals other than bees, typically large animals dangerous to humans. This is interpreted by some human cultures as punishment for prior failure to reward the bird, and by others as an altruistic warning behavior. Here, we present quantitative evidence from hundreds of honeyguide‐human interactions in Mozambique of greater honeyguides guiding humans to snakes (*n* = 3) and a dead mammal (*n* = 1). We show that guiding behavior to these vertebrates was (i) spatially and acoustically analogous to honeyguide behavior when guiding to bees, (ii) did not occur more frequently after not being rewarded with beeswax by humans, and (iii) was rare (3.7% of human‐honeyguide interactions in 1 year; 0% in others). We review historical accounts and cultural explanations for this behavior and use these to inform five hypotheses for why honeyguides guide people to nonbee animals. Our field data were most consistent with the hypothesis that guiding to nonbee animals results from a cognitive recall error of spatial information. We suggest that this behavior is unlikely to function as punishment, yet may coincidentally benefit honeyguides over longer timescales by initiating a human cultural interpretation that reinforces human cultural traditions of rewarding honeyguides with beeswax.

## Graphical abstract

We show that greater honeyguides guide humans to nonbee destinations (snakes and a dead mammal); yet this is a rare occurrence, happening in only 3.7% of human‐honeyguide interactions in 1 year and 0% in other years. We show that guiding behavior to these vertebrates is spatially and acoustically similar to honeyguide behavior when guiding to bees; yet it does not more frequently follow rewarding behavior by humans (leaving behind a beeswax food reward) than nonrewarding behavior by humans (humans do not harvest a bees' nest after being guided to it).

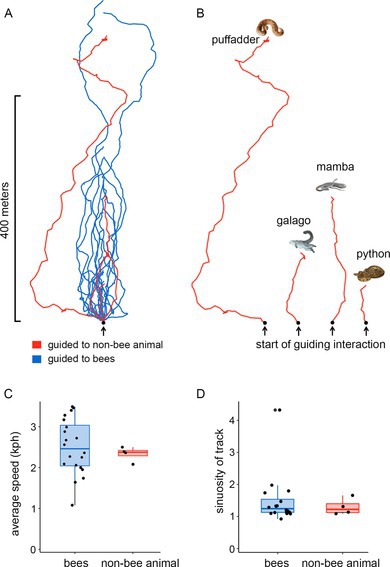

## 1 Introduction

**FIGURE 1:**
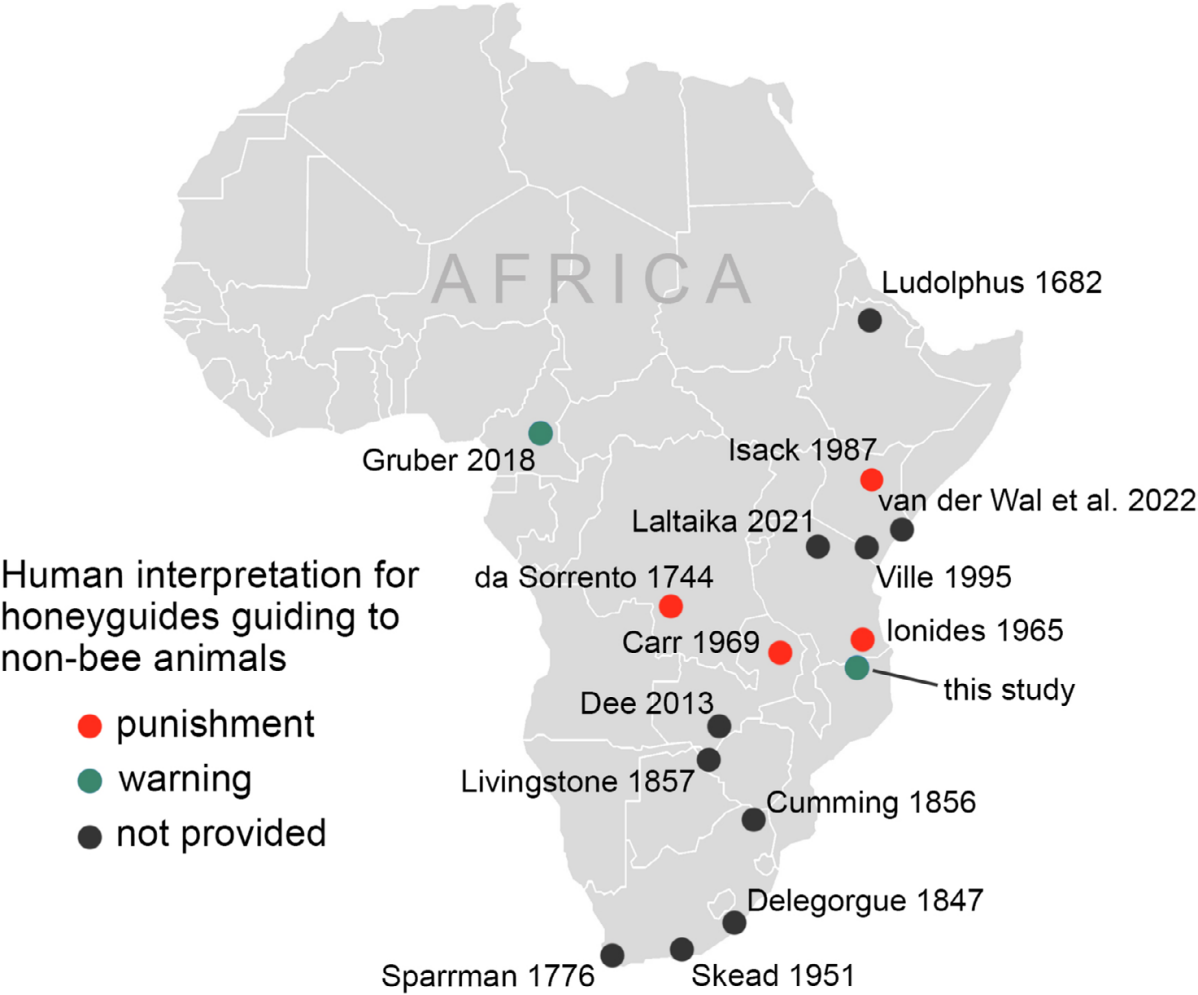
Published accounts from 14 African honey‐hunting cultures of humans being guided by honeyguides to nonbee animals, with cultural explanations (where provided) for this behavior. Direct quotes from the literature are provided in Appendix 4.

Mutualistic species partnerships are not always beneficial to both partners all the time (Bronstein 2001). One partner may fail to provide the reward that the other expects (Clutton‐Brock and Parker 1995), causing the costs of engaging in mutualistic behavior to outweigh its benefits (Riehl and Frederickson 2016). Across sub‐Saharan Africa, greater honeyguide birds (hereafter “honeyguides”) guide humans to bees' nests, exchanging their knowledge of the locations of bees' nests for a food reward (beeswax) resulting from human honey hunters' abilities to subdue bees with smoke and open their nests using axes (Friedmann 1955; Isack and Reyer 1989). In this mutualism, one partner occasionally fails to do their part: humans may fail to harvest the nest or reward the bird, and a guiding honeyguide sometimes fails to show a human a bees' nest (Isack 1987; Spottiswoode et al. 2016; Wood et al. 2014). The latter can occur when a honeyguide prematurely stops guiding before any destination is reached. However, according to centuries of African Indigenous accounts (Figure 1), failure to find bees can also occur when a honeyguide deliberately guides humans to an animal dangerous to humans, such as a lion, buffalo, elephant, rhino, large venomous snake, or occasionally a carcass (Friedmann 1955; Isack 1987).

The first account in scientific literature of honeyguides guiding humans to such nonbee species dates from 1776 when Khoe‐Sān honey hunters in the Western Cape of South Africa recounted being occasionally guided to “dangerous animals” (Sparrman 1777). Seventy‐nine years later, David Livingstone attempted to evaluate the frequency of such behavior by asking East African honey hunters whether they had been guided by a honeyguide to anything but a bees' nest and reported that “Only one out of the 114 could recall that he had had such an experience although all had been guided on a great many occasions” (Livingstone 1858). Later, in his landmark study of honeyguides in Kenya, Hussein Isack reported that “A common belief that the Boran share with several other communities in Africa is that the bird often guides man to animals such as lions, buffalos, elephants, rhinos, large poisonous snakes or to enemies or murderers from a hostile tribe… It is believed that the bird resorts to this abnormal behavior as a result of man's failure to spare some food for it after the bird had helped him discover a [bee] colony” (Isack 1987).

Unfortunately, few published accounts of guiding to nonbee animals across Africa include first‐hand observations, and the ability of honeyguides to deliberately guide to nonbee species has typically been treated with skepticism by both ornithologists (Friedmann 1955; Short and Horne 2001) and by some honey hunters (Isack 1987). Such skepticism is understandable given the temptation of observers to attribute incidental encounters with such animals as deliberate on the part of the honeyguide. It is nonetheless striking that reports of this phenomenon are widespread across Africa (Figure 1) (Isack 1999).

If honeyguides deliberately guide people to nonbee animals, it is not known why such behavior would occur and whether it is adaptive. Human cultural interpretations for such a behavior vary culturally across Africa (Appendix 4), with the most influential interpretation being that honeyguides do so to punish a person for prior failure to reward them with beeswax following a honey harvest (Carr 1969; da Sorrento 1744; Friedmann 1955; Ionides 1965; Isack 1987). This has, to our knowledge, only been documented from four honey‐hunting cultures (Figure 1), but strongly appeals to the human imagination and is widely repeated. Evidence for punishment between heterospecifics, where one partner sacrifices payoffs from interaction to harm a cheating partner, has rarely been observed among nonhuman animals (Jensen 2010; Raihani and Bshary 2019; Raihani et al. 2012), yet is known from client‐cleaner fish mutualisms (Bshary and Grutter 2005).

In contrast, some honey‐hunting cultures (including the Yao culture which is the focus of the present study) believe that when honeyguides guide a human to animals other than bees, the honeyguide is acting altruistically by warning a human of a danger nearby (Gruber 2018; D.J.L, M.M., and C.N.S unpubl. data). Meanwhile, other cultures do not provide a clear functional interpretation for this behavior and accept that “bad honeyguides” rarely yet deliberately guide people to animals other than bees (Isack 1999). Some writers dismiss the idea that honeyguides guiding to dangerous animals or other objects is intentional and instead consider these encounters to be chance events en route to a bees' nest (Friedmann 1955; Short and Horne 2001). Across a wide variety of accounts, what remains consistent is the reported infrequency of honeyguides guiding to nonbee animals, even in areas where honeyguide‐human cooperation is common (e.g., Isack 1987; Livingstone 1858). Infrequent observations of an apparently rare behavior would also have likely reduced further as human‐honeyguide cooperation has diminished across the African continent (Isack 1999; van der Wal et al. 2022a).

In this study, we present recent quantitative data from interactions in Mozambique between Yao honey hunters and honeyguides, which corroborate historical accounts that honeyguides deliberately guide humans to nonbee animals. We review five biological hypotheses (Table 1), some of which draw on human cultural explanations for why honeyguides may guide to nonbee animals, and generate testable predictions for each. Finally, prompted by the perspectives of Yao honey hunters and our firsthand observations, we use additional field data to retrospectively evaluate certain predictions of each hypothesis.

**TABLE 1:** Five hypotheses, with associated predictions, for why honeyguides guide humans to the location of nonbee animals.

Predictions	Hypotheses				
	(i) Incidental encounter	(ii) Punishment	(iii) Warning	(iv) Mobbing	(v) Spatial recall error
Honeyguides stop guiding at nonbee animals, like at bees	−	+	+	+	+
Guiding to nonbee animals tends to occur after a nonrewarded interaction	na	+	−	na	na
Nonbee animal is dangerous to humans	na	+	+	na	na
Nonbee animal is dangerous to honeyguides	na	na	na	+	na
Honeyguides slow down and stop when arriving at nonbee animals, like at bees	−	+	+	+	+
Honeyguides make the same calls when arriving at nonbee animals, as they do at bees	−	+	+	?	+
Honeyguides mob the species to which they guide humans	na	na	na	+	na
Nonbee animal is alive	na	+	+	+	na
Nonbee animal is spatially predictable (i.e., immobile, or returns to the same location at predictable times)	−	+	+	+	+

*Note:* (i) a null hypothesis that these are *incidental encounters* en route to a bees' nest, or that it is deliberate and (ii) functions as *punishment* of nonreciprocating humans, (iii) functions as a cooperative *warning* signal to humans, (iv) functions to recruit *mobbing* partners, or (v) results from a cognitive *spatial recall error* regarding the destination of the guiding event. Positive and negative symbols indicate whether or not each hypothesis makes this prediction, and “na*”* indicates that a prediction is not relevant to the hypothesis. Green cells indicate empirical support for the designated prediction, orange cells indicate no support, and yellow cells indicate mixed support.

## 2 Material and Methods

### 2.1 Study Site

This study was conducted in a 280 km^2^ area within the Niassa Special Reserve in northern Mozambique (Lloyd‐Jones et al. 2022; Spottiswoode et al. 2016). This area is inhabited and utilized by honey hunters of the Yao ethnic group who live in Mbamba village (12°12’S, 38°01′ E), which has a population of ca. 2000 inhabitants, including > 20 professional honey hunters (Lloyd‐Jones et al. 2022). In this miombo woodland area, honeyguide–human interactions occur daily under conditions likely comparable to those under which the mutualism evolved (see Cram et al. 2023; Lloyd‐Jones et al. 2022; Spottiswoode et al. 2016 for further details of the study system). Yao honey hunters consistently reward honeyguides with beeswax of a varying amount following a harvest (Lloyd‐Jones et al. 2022), although the act of harvesting a bees' nest can also enable a honeyguide to benefit from wax via access to the tree cavity containing leftover beeswax. Yao honey hunters do not reward honeyguides when they are guided to a bees' nest that they do not harvest. This rewarding behavior of Yao honey hunters is directly linked to a belief that failure to do so results in honeyguides being less cooperative in the future, both by guiding to fewer bees' nests and by guiding to other dangerous animals instead.

### 2.2 Data Collection

Given that guiding to nonbee animals appears to be rare, it was not possible to design a study specifically documenting it. Rather, we collected data opportunistically during other studies and experiments with honeyguides.

Four datasets are used here. First, four first‐hand accounts of honeyguides guiding honey hunters to nonbee animals were recorded in 2018 from separate honey hunts under natural conditions and included visual, spatial, and in some cases, acoustic data. We used these data to describe our direct observations of honeyguides guiding to nonbee animals. Second, 108 records of honey hunts (defined as a single human‐honeyguide interaction with a unique starting point) between January 28 and December 22, 2018. We used these data to calculate overall rates of guiding to bees' nests compared to nonbee animals. Third, we used a subset of 24 honey hunts (from the 108 honey hunts) for which we have complete GPS tracks of guiding interactions, and a further subset of 22 honey hunts with audio recordings of honeyguide vocalizations, recorded between May 18 and June 27, 2018. Twenty of these honey hunts ended with the honey hunter finding bees, and four ended with the honey hunter finding an animal other than bees. We used the 24 GPS tracks to analyze spatial movement patterns and used the 22 audio recordings to analyze honeyguide vocalizations while guiding. Some of these honey hunts were recorded on the same day (mean number of honey hunts per day ± SE = 1.71 ± 0.29, *n* = 24), but when this was the case, the starting points were < 1500 m apart, making it likely that different honeyguide individuals were involved. In the last dataset, we selected 28 honey hunts that occurred in close (< 1500 m) proximity to each other within a 7‐day period. We used these interactions to calculate rates of honeyguide behavior in relation to prior rewarding behavior by humans (i.e., provision of beeswax) by humans.

Although we have conducted research at this site since 2013, we only analyzed data from 2018, given that all our observations of being guided to nonbee animals were made in this year, and we wanted to ensure that environmental conditions were comparable for guiding events with different outcomes.

To initially locate the bees' nests, one researcher (D.J.L or C.N.S) accompanied two Yao honey hunters (one of whom is a coauthor: M.M) on a honey hunt as they elicited guiding behavior from greater honeyguides using culturally determined, locally specialized calls, given either naturally or using controlled speaker playbacks (repeated loops of either the locally specialized call or, as a control treatment, a human calling his name) as part of behavioral experiments following methods in Spottiswoode et al. (2016). Honey hunts involving such playbacks contributed one of the four instances of guiding to dangerous animals, and six of the 28 guiding events contributed to our post hoc analyses of whether nonrewarding behavior by honey hunters is more likely to precede being guided to nonbee animals. In these seven instances, the playback was either a repeated loop of a human calling their name (*n* = 3), used as a control sound, or a specialized “brrr‐hmm” sound (*n* = 4). All honey hunts began at intervals along vehicle tracks at least 500 m apart, and at least 1500 m apart when conducted on the same day, to reduce the confounding effect of prior interactions with honeyguides.

For spatial analyses of guiding behavior to bees' nests or nonbee animals, we use GPS tracks of 24 guiding events (points recorded at set 1 s intervals using Garmin eTrex 30, Garmin USA), which all involved natural honey hunter calls to honeyguides, except one guiding event to a puffadder which was accompanied by control playbacks (described above). The final destinations of all guiding events (whether bees' nests or other animals) were previously unknown to us or the honey hunters.

For audio analyses of honeyguide calls, we used stereo audio tracks recorded on 22 of these events (two of which involved guiding to nonbee animals) by D.J.L, using a unidirectional Sennheiser ME66 (Sennheiser Inc., Wennebostel, Germany) microphone recording to a Sony PCM‐M10 (Sony Group Corporation, Tokyo, Japan), continuously aimed at the honeyguide. Acoustic recordings began prior to guiding and continued until the bees were located. All audio tracks were saved as Linear PCM (WAV) files at 96.00 kHz/24 bit and were reviewed, clipped to length, and normalized to −3.0 dB using Audacity version 3.0.5 (Audacity Team 2022). Raven Pro v. 1.6.1 (Center for Conservation Bioacoustics Cornell 2019) was then used to display sounds as spectrograms, from which we manually selected bounding boxes for individual syllables from within “chatter” (Audio 1) and “indication” (Audio 2) call types (Isack 1987). Following Isack (1987), we distinguished between typical “chatter” calls given by honeyguides while guiding and “indication” calls, which are also sometimes given while guiding but characteristically given as the honeyguide approaches the bees' nest, prior to going silent (Appendix 3, Figure A2).

**AUDIO 1:** Typical 'chatter' calls made by greater honeyguides when they initiate guiding and throughout a guiding interaction with a honey hunter. Audio content can be viewed at https://onlinelibrary.wiley.com/doi/10.1002/ece3.71136

**AUDIO 2:** Typical 'indication' calls made by greater honeyguides which are often (but not always) produced as they arrive at a bees' nest or nonbee animal, prior to going silent. Audio content can be viewed at https://onlinelibrary.wiley.com/doi/10.1002/ece3.71136

### 2.3 Data Analyses

We tested whether honeyguides were more likely to guide humans to nonbee animals after not being rewarded by humans (i.e., when a human follows a honeyguide to a bees' nest but does not harvest; Figure 4) by focusing on 11 honey hunts that had been preceded by two or more prior honey hunts within 1500 m or less, within the previous 7 days. In total, there were 28 prior honey hunts (two were counted twice, due to their proximity in different directions to two different harvests). For each of the 11 focal honey hunts, we calculated the proportion of prior honey hunts which resulted in a bees' nest harvest and consequently a wax reward versus finding a bees' nest but choosing not to harvest (Figure 4). We next compared the proportion of prior rewards for focal honey hunts that involved being guided to nonbee animals (4 of the 11 focal honey hunts), versus honey hunts that involved being guided to bees' nests (7 of the 11 focal honey hunts). We ran a generalized linear model (GLM) with a binomial logit link function to test the effect of prior reward rates on the binary outcome of being guided to a bees' nest or to nonbee animals. We visualized residuals and plotted them against fitted values to assess model assumptions, which indicated no severe deviations from linearity on the logit scale, no strong outliers, and approximate homoscedasticity.

We selected the < 1500 m and 7‐day limits for the above dataset under the assumption that repeated interactions with the same individual honeyguide were possible within such limits, although it is plausible that many honeyguides could have been involved in prior interactions, given high honeyguide densities at this site (Cram et al. 2023). This represents an imperfect best attempt at detecting a relationship between past rewarding behavior and guiding to nonbees, with the parameter values supported by past work at this location showing that honeyguides can move over an area with a radius of at least 1500 m over short time periods (Spottiswoode et al. 2016).

Three measures of human movement were extracted from GPS tracks of honey hunters following honeyguides: distance guided, speed, and sinuosity. For distance guided, start points were determined from the simultaneous audio recordings as to where the honeyguide arrived and first “chattered” to the human, and stop points as to where the honeyguide reached either a bees' nest or a nonbee animal. Speed was measured from the GPS track of the honey hunter carrying a GPS. The sinuosity of human movement was the ratio of actual distance traveled to the distance of the shortest as‐the‐crow‐flies path, measured using GPS tracks (Wood et al. 2021).

We used R v. 4.0.3 (R Core Team 2023) for all statistical analyses. To test whether the distance guided to nonbee animals was statistically different from that when guided to a bees' nest, we used a Mann–Whitney U test because as the data violated assumptions of normality and homogeneity of variances (tested using Shapiro–Wilk and Levene's tests). Similarly, to test whether average speed while being guided to a bees' nest differed from that while being guided to nonbee animals, we used a Wilcoxon rank‐sum test because these data solely violated the assumption of normality (assessed using Shapiro–Wilk and Levene's test for homogeneity of variance). To test for differences in the sinuosity of tracks ending at nonbee animals compared to those ending at bees' nests, we used a nonparametric Mann–Whitney U test because preliminary Shapiro–Wilk and Kolmogorov–Smirnov tests indicated that these data violated the assumptions of normality and homogeneity of variance distributional assumptions.

To analyze acoustic differences between honeyguide “chatter” and “indication” calls, we used a principal components analysis (PCA) using the “stats” package in R (Bates et al. 2015), based on 20 acoustic variables for each of the “chatter” and “indication” syllables (details in Appendix 3, Table A1) extracted using the “warbleR” package (Araya‐Salas and Smith‐Vidaurre 2017).

### 2.4 Ethical Note

No animals were captured for the purposes of this study. Free‐living greater honeyguides “opted in” to cooperative behavior with humans by guiding them to wild bees in a natural setting. Birds and humans were audio recorded with minimal interference. This research was approved by the University of Cape Town Faculty of Science Research Ethics Committee, Permit number FSREC 50–2018, and data were collected with permission from Administração Nacional das Áreas de Conservação (ANAC), Mozambique, Permit nos. 008/2015, 11/11/2016, 15/2019, 09/2020, and 04/08/2022.

## 3 Results

### 3.1 Honeyguides Only Rarely Guide to Animals Other Than Bees

At our study site in northern Mozambique, the observed rate of being guided to animals other than bees in 2018 was 3.7% (4 of 108 guiding events leading to a distinct destination). It was 0% in D.J.L and C.N.S's own personal experience of guiding events leading to a distinct destination (i.e., were “successful”) at this location during 2013–2017 and 2019–2024 (approximately *N* = 350), and M.M also experienced no other events during this period as an active honey hunter (approximately *N* = 330 honey hunts during 2017–2022).

### 3.2 Direct Observations of Honeyguides Guiding to Nonbee Animals

**FIGURE 2:**
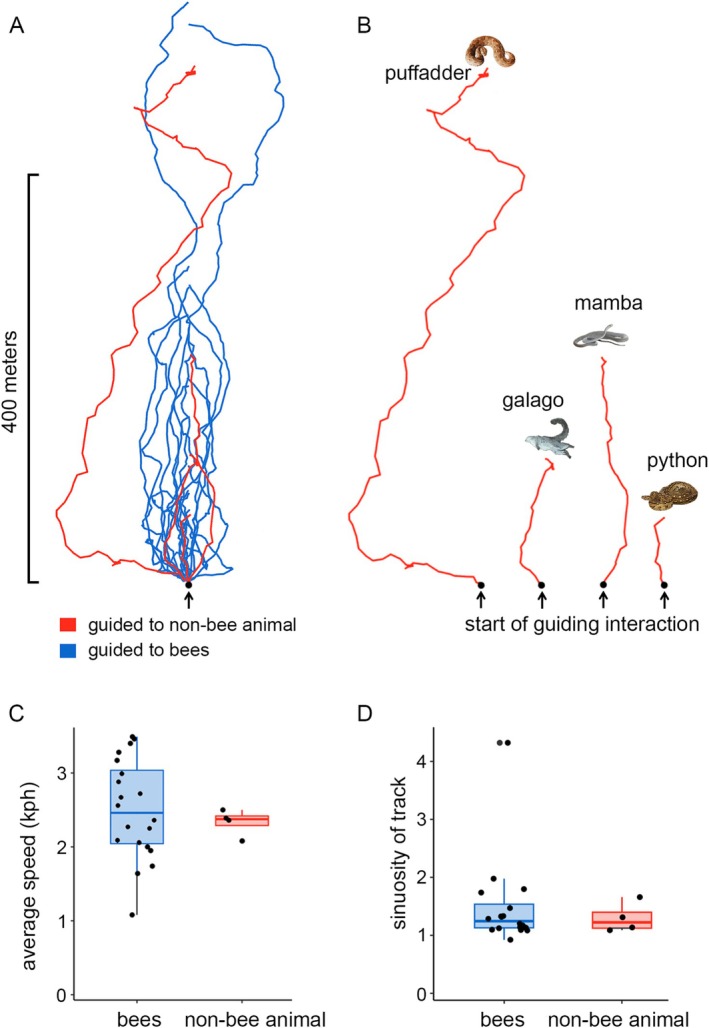
(A) GPS tracks of 20 guided honey hunts ending at bees' nests versus four guided honey hunts to nonbee animals (all tracks rotated to align along a straight vertical line between their start and end points). (B) GPS tracks of four guided honey hunts to nonbee animals plotted to scale but all oriented vertically. (C) Average speed (kph) of guided honey hunts to bees' nests and nonbee animals. (D) Sinuosity (the ratio between GPS distance and straight‐line distance) for honey hunts to bees' nests compared to honey hunts ending at nonbee animals.

**FIGURE A1:**
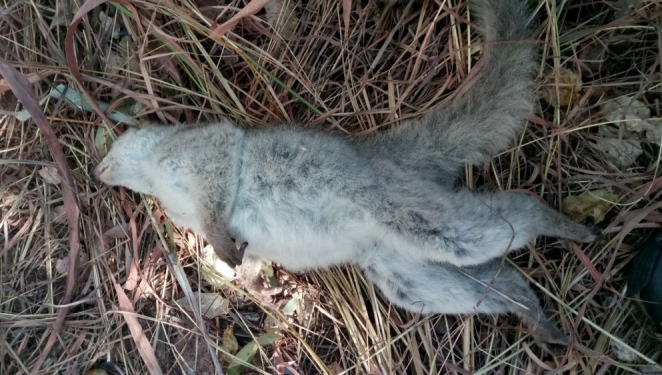
Dead greater galago ( *Otolemur garnettii* ), to which the authors were guided by a honeyguide on June 13, 2018 (photograph by D.J.L).

**FIGURE A2:**
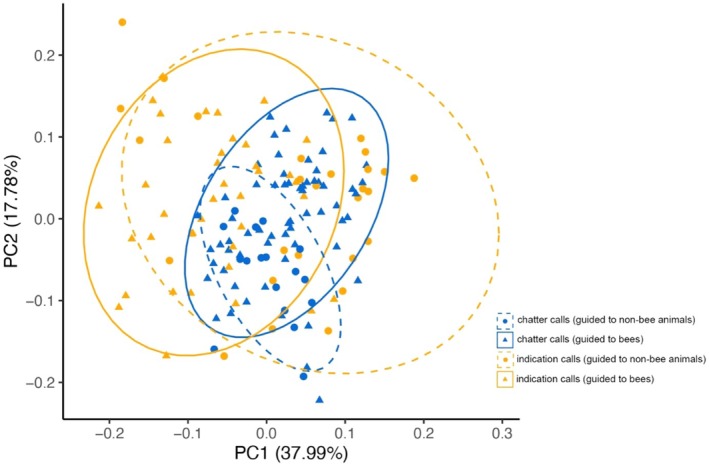
Results of a principal components analysis (PCA) comparing the acoustic parameters of two types of honeyguide sounds given while guiding: (1) “chatter” calls typically given by the honeyguide when guiding to a bees' nest (blue points with ovals indicating 95% confidence levels), and (2) “indication” calls given by the honeyguide as they near the location of a bees' nest when guiding (yellow points with lines indicating 95% confidence levels). Honeyguide “chatter” and “indication” calls are generally distinctive from each other, with some syllables of the “chatter” calls generally intermingled with distinctive squeaky “indication” syllables (Isack 1987). There is a high degree of overlap between “chatter” calls en route to bees' nests, and en route to animals other than bees (blue points). These are somewhat separate to “indication” calls, which also overlap regardless of whether they are given en route to bees' nests or en route to animals other than bees (yellow points). All recordings were made in the course of this study; datapoints are individual syllables from the calls of *n* = 6 honeyguides en route to bees' nests, and *n* = 2 honeyguides en route to animals other than bees.

We were guided by a honeyguide to a nonbee animal on four occasions:

1. On May 18, 2018, M.M, C.N.S, and one other honey hunter (Issufo Mussa) were guided by an adult female honeyguide while conducting a playback experiment (details in Methods). Guiding began at 09:05 h (all times are local time: UTC + 2) and continued until 09:26 h when the honeyguide went silent (as is typical behavior signaling arrival at bees; Isack and Reyer 1989) near an adult puffadder (*Bitis arietens*) in short grass. We followed the honeyguide for an 838 m walking distance (Figure 2), during which the honeyguide vocalized with “chatter” calls and at the destination flew with low swooping flight in the same way as when guiding to bees. This interaction was not audio recorded.
2. On June 13, 2018, M.M, D.J.L, and one other honey hunter (Orlando Yassene), during a completely natural honey‐hunt (i.e., one in which all interaction decisions and vocalizations were being made by Yao honey hunters), were guided by an adult male honeyguide to a bees' nest. On arrival at the bees' nest, the honeyguide went silent. After 65 s, the honeyguide began calling again and guided our human party onward, at 08:40:10 h, to what we presumed was another bees' nest, given that honeyguides commonly lead humans to multiple bees' nests in succession (Friedmann 1955; Isack 1987; Spottiswoode et al. 2016). The honeyguide flew ahead of the honey hunter party, chattering continuously, stopped, and then doubled back ca. 10 m, perching in a tree adjacent to a dead greater galago ( *Otolemur garnettii* ) splayed out in short grass at 08:44:00 h (Appendix 1, Figure A1). Audio recordings of this honeyguide were very faint due to background noise and were therefore not used in acoustic analyses. The galago appeared intact and was unlikely to have been there for more than 24 h, showing no sign of decomposition or being scavenged. The honeyguide remained silently perched in adjacent trees for the full duration we were there (ca. 10 min).
3. On June 18, 2018, while recording honey‐hunting audio signals during another natural honey‐hunt, D.J.L and two Yao honey hunters (Rui Francisco, Aloisi Chole Sindi) were guided by an adult female honeyguide (starting 10:53:59 h) directly to an adult black mamba ( *Dendroaspis polylepis* ) at 11:00:16 h. The snake was coiled up and basking on a termite mound. Throughout, the honeyguide showed all the characteristic behaviors of guiding to bees: a loud chattering call initially, strong continuity of chattering to humans, who were responding using traditional “brrr‐hmm” signals, staying within sight 15–25 m ahead of the party, and a change from a “chatter” call to the “indication call” (a signal observed by us, and widely reported by honey hunters, to be given when nearing a bees' nest; Isack 1987) 1 m 15 s before arriving at the snake (Video 1). The honeyguide flew low over the snake and perched above it, in the same way as near a bees' nest, and went immediately silent (Video 1). When we approached within ca. 10 m, the snake disappeared into the termite mound. We systematically searched (radius 20 m) for bee's nests for ca. 10 min, but we found none. Throughout this search, the honeyguide remained silently perched nearby, first in the tree above the snake and then in adjacent trees.
4. On June 22, 2018, while recording honey‐hunting audio signals during a natural honey‐hunt, D.J.L and two Yao honey hunters (Carvalho Issa and Armando Pita) were guided by an adult male honeyguide to a southern African rock python ( *Python natalensis* ) basking at the base of a tree. Within 10 s of arriving within 5 m of the snake, it went into a cavity at the base of the tree. The honeyguide had guided us 5 min earlier to a bees' nest (which the honey hunters decided not to harvest), then resumed chattering and flew onwards at 10:46:22 h. The party followed the honeyguide for 75 m and arrived at the python at 10:48:34 h. The honeyguide made “indication” calls as it flew into the tree above the python and then perched silently 6–7 m above the hole in the tree the python went into. On this occasion, as with the three preceding records, the Yao honey hunters reported at the time that they were certain that they had been guided to this location deliberately by the honeyguide.

**VIDEO 1:** Audio recording and GPS tracks from a honeyguide‐human cooperative interaction in which the greater honeyguide guided a group of three people (D.J.L and two Yao honey hunters) to a black mamba (*Dendroaspis polylepis*) basking on a termite mound. Video content can be viewed at https://onlinelibrary.wiley.com/doi/10.1002/ece3.71136

On other occasions, we have encountered numerous snakes, and occasionally elephant ( *Loxodonta africana* ), buffalo ( *Syncerus caffer* ), and lion ( *Panthera leo* ) while being guided by honeyguides, but we consider these to be incidental encounters since in most cases it was clear from the honeyguide's behavior that it was leading us beyond these animals. On one occasion our interpretation differed from that of the honey hunters we accompanied: on June 11, 2023 we encountered a Mozambique spitting cobra ( *Naja mossambica* ) while being guided, and the honey hunters we accompanied reported post hoc that the honeyguide had deliberately guided us to the snake. However, the honeyguide did not pause or cease calling at the location where the snake was sighted, and continued to guide us to a bees' nest, so we consider this a false positive (Appendix 2).

### 3.3 Honeyguide Spatial Behavior When Guiding to Nonbee Animals Is Similar to When Guiding to Bees

Based on the subset of tracks with high‐resolution GPS data, the distance over which honeyguides guided us to nonbee animals (range: 75–838 m, mean ± SE = 329 ± 173 m, *n* = 4) was not significantly different (Mann–Whitney U test; W = 36, *p* = 0.79, *n* = 24) from the distance over which honeyguides guided us to bees (range: 73–890 m, mean ± SE = 308 ± 47 m, *n* = 20). Walking speed when being guided to nonbee animals (range: 2.08–2.50 kph, mean ± SE = 2.3 ± 0.09 kph, *n* = 4, Figure 2C) was also not significantly different (Wilcoxon rank sum test; *n* = 4, 20, W = 35.5, *p* = 0.76) from walking speed when being guided to bees (range: 1.08–3.49 kph, mean ± SE = 2.5 ± 0.15 kph, *n* = 20). Lastly, the sinuosity of the GPS tracks recorded en route to nonbee animals (range: 1.09–1.66, mean ± SE = 1.30 ± 0.13, *n* = 4) was not statistically different (Mann–Whitney U test; *n* = 24, W = 46, *p* = 0.68) from track sinuosity when guided to bees (range: 0.92–6.57, mean ± SE = 1.71 ± 0.30, *n* = 20).

### 3.4 Honeyguides Emit the Same Call When Nearing Nonbee Animals as when nearing bees

**FIGURE 3:**
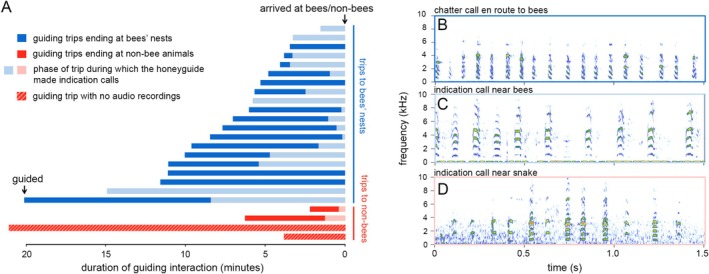
(A) Graphical representation of the overall duration and call‐types given by honeyguide over 24 GPS‐recorded honey hunts, of which 22 were audio recorded. Change in tone from dark blue or red to light blue or red indicates the transition in “chatter” guiding calls to “indication” guiding calls, which are typically used in closer proximity to bees (Isack 1987). Spectrogram of: (B) typical chatter call given by a honeyguide en route to a bees' nest, (C) “indication” calls when guiding to bees, and (D) “indication” calls when guiding to a black mamba snake.

To test whether honeyguides produce similar calls when guiding honey hunters to bees as to nonbees, we used audio recordings from two instances of guiding to nonbees and from 20 instances of guiding to bees' nests. On both of the former, the honeyguide altered its calls from the “chatter” call (given initially upon guiding and over much of a honey‐hunt) to “indication” calls (Figure 3). This switch in call type was observed in 17 of 20 (85%) audio‐recorded events of guiding to bees' nests (Figure 3A). The mean straight‐line distance (from the destination) at which the “indication” calls started to be produced when being guided to bees was mean ± SE = 63 ± 15.4 m, *n* = 16, and when being guided to snakes was mean ± SE = 16 ± 1 m, *n* = 2). On all four occasions of being guided to nonbees, the honeyguide went silent upon arrival at that location, and in three of four instances perched within sight in an adjacent tree in the same way as when guiding to bees (pers. obs. all authors, also Isack 1987; Short 1988; Short and Horne 2001).

### 3.5 Guiding to Nonbee Animals Is no More Likely After Nonharvests by Humans

**FIGURE 4:**
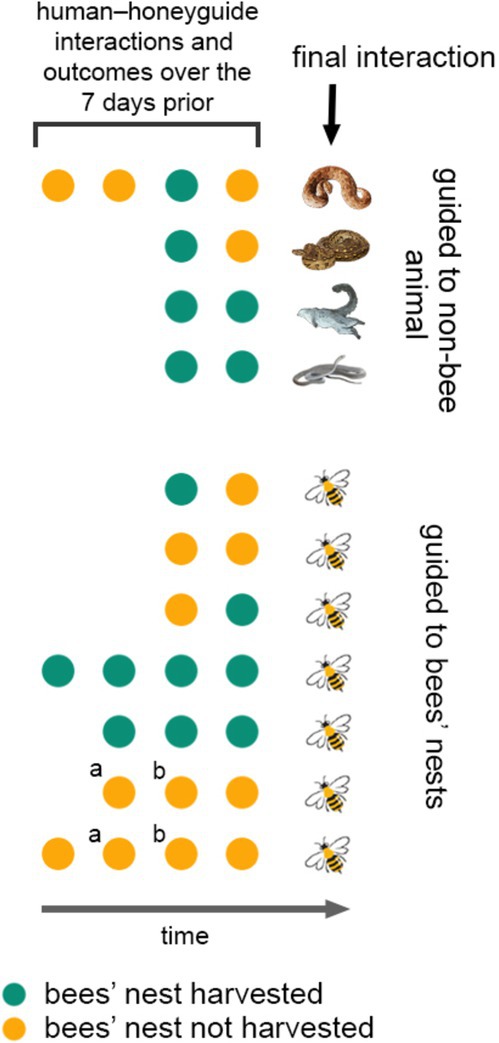
Guiding to nonbee animals does not appear to be more likely to occur in areas where humans were recently guided to a bees' nest but did not harvest (resulting in no wax reward for a honeyguide). In this graphical summary, we show patterns of human‐honeyguide interactions and harvests (and therefore wax rewards to honeyguides), over 7 d, in 11 subsets of our study area, in relation to being subsequently guided to a nonbee animal (*n* = 4; animals illustrated are, from the top, puffadder, southern African rock python, greater galago, black mamba), or to a bees' nest (all *Apis mellifera* ; *n* = 7). Harvests denoted *a* and *b* are recorded twice for two different interactions with honeyguides.

As an imperfect test of whether honeyguides were more likely to guide humans to nonbee animals following nonrewarding behavior by humans, we compared the proportion of honey hunts in the same area that were rewarded prior to being guided to nonbee animals versus prior to being guided to bees' nests (Figure 4). Honeyguides guided us to animals other than bees following prior reward rates of 0.5, 0, 0.75, and 0 (Figure 4), and to bees' nests following prior rewarding rates of 0.5, 1, 0.5, 0, 0, 1, 1 (Figure 4). While recognizing that the dataset is small, we found no significant effect of prior rewarding rates on the likelihood of being guided to nonbee animals compared to bees (Generalized Linear Model, Estimate = −1.69, SE = 1.71, z = −0.99, *p* = 0.32).

## 4 Literature Review and Five Hypotheses for Guiding Nonbee Animals

We found 20 published references to honeyguides guiding humans to nonbee animals, originating from nine countries (original quotations and sources are given in Appendix 3, Table A1, and geographical locations, dates, and interpretations are summarized in Figure 1). Of these 20 published references, five directly state the local human cultural interpretation for why honeyguides guide to nonbee animals (four are punishment, one is an altruistic warning). We do not consider in depth here the hypothesis that honeyguides may benefit from guiding humans to dead animals or carcasses, either because they benefit from the insects on the carcass (Delegorgue 1847) or because the flies at a carcass are a bee‐like stimulus (Friedmann 1955). This is because we have neither observed nor found any reports of honeyguides feeding on carcasses or flies at carcasses. In Table 1 we present five hypotheses explaining this behavior, together with their testable predictions. Hypotheses (1) to (3) have been previously proposed (da Sorrento 1744; Friedmann 1955; Isack 1987; Sparrman 1777) whereas (4) and (5) are proposed here for the first time. Briefly, these hypotheses are that guiding to animals other than bees is (1) the result of an *incidental encounter* occurring en route to a bees' nest, which humans mistakenly perceive to be the destination; (2) *punishment* of nonrewarding humans; (3) an *altruistic warning* to rewarding humans; (4) *recruitment* of mobbing partners; and (5) a *cognitive recall error* relating to how honeyguides store spatial information about memorable objects in the same way as they do bees' nests.

## 5 Discussion

In this study, we present first‐hand data supporting centuries of assertions by a range of African honey‐hunting cultures (Figure 1) that honeyguides rarely but intentionally guide honey hunters to animals other than bees. Honeyguides guided us to three snake species and a dead mammal, in each case stopping at the animal and producing “indication” calls, in the same way as when a honeyguide has arrived in the vicinity of a bees' nest. Furthermore, on three of the four observations of guiding to nonbee animals, honeyguides flew low over the snakes which, when given at bees, is a cue of arrival at a bees' nest located low down (Isack 1987 and pers. obs. all authors). The track sinuosity, distance followed, and timing of when honeyguides produced “indication” calls prior to arriving at nonbee animals were all within natural ranges observed when guided to bees (Figure 4). While the small sample size means we have to interpret these findings with caution, taken together, they are inconsistent with the first hypothesis, that encounters with these animals are incidental occurrences en route to bees (hypothesis i, Table 1).

Next, we cautiously evaluate our field data against three functional hypotheses for why honeyguides deliberately guide humans to animals other than bees: as punishment, as an altruistic warning, or to recruit mobbing partners.

First, if punishment toward humans for nonrewarding behavior is adaptive for honeyguides, then being guided to nonbee animals rather than bees should follow nonrewarding behavior by humans, and encounters with these nonbee animals should have the potential to negatively impact the human(s). Neither of these predictions was supported: the degree to which honeyguides (not necessarily the same individual) in the vicinity had previously received a beeswax reward for cooperative behavior did not predict whether we were subsequently guided to bees or nonbees, albeit in a small sample with weak statistical power (Figure 4C). In one instance, a honeyguide guided us to bees after taking us to a python, and we were guided to a dead galago immediately after being guided to and harvesting a bees' nest. Finally, the nonbee animals were largely nonthreatening to humans, since Yao honey hunters regularly encounter snakes, and even the black mamba, an agile and feared species, attempts to evade humans as a first response (Spawls et al. 2018), which is considered by Yao honey hunters to be far less threatening than an unexpected encounter with a large mammal such as buffalo, elephant, and hippopotamus.

Further to this empirical evidence, there are theoretical objections to the evolution of punishment by honeyguides. Punishment would require individual birds to: (a) recognize cheating individual honey hunters, (b) have repeat encounters with these cheating individuals, and (c) cause humans to alter their behavior to be more cooperative in future encounters (Raihani et al. 2012). These conditions are unlikely to be met, given that honey hunters interact with many different honeyguides within the large area (> 812 km^2^) over which each individual honey hunter ranges likely with a low re‐encounter rate (unpubl. data D.J.L and C.N.S; Cram et al. 2023). Moreover, certain other honey‐hunting cultures elsewhere in Africa intentionally withhold wax rewards from honeyguides, by either pretending not to spot bees' nests or choosing not to harvest so that the honeyguides “stays hungry” and shows them more bees' nests; yet there is no evidence that honeyguides are less likely to cooperate with, or more likely to “punish,” humans in these populations (e.g., Isack 1987; Laltaika 2021; van der Wal et al. 2022b; Wood et al. 2014).

Even in cultures that deliberately reward honeyguides from harvested bees' nests (such as the Yao culture in this study), honeyguides often experience unintentional nonrewarding behavior. For example, in the miombo woodland habitat of our study area, a tree containing a bees' nest can sometimes be either too big to harvest or too hard to cut into with an ax, or the bees' nest may appear to not contain enough honey to merit the effort (all authors, unpubl. data). The cost to humans of depriving honeyguide partners appears to be low, since honeyguides continue to cooperate regularly across varied cultural settings (Laltaika 2021; van der Wal et al. 2022a; Wood et al. 2014). Furthermore, unlike in other systems of punishment (Raihani et al. 2012), in the honeyguide –human system there is no scope for coercion: neither humans nor honeyguides can force their cooperative partners to cooperate. We conclude that there is neither empirical nor theoretical support for a punishment hypothesis of guiding to nonbees.

Second, in contrast to a punishment hypothesis, some cultures interpret being guided to dangerous animals as a form of *altruistic warning* to the humans of danger ahead (hypothesis iii, Table 1). These cultures include the Yao honey‐hunting community, with whom we collaborate in this study (pers comms., all authors), and at least some other cultures (Gruber 2018). This hypothesis makes certain similar predictions to those for punishment (i.e., that honeyguides should guide deliberately to living but spatially predictable animals dangerous to humans). However, unlike the punishment hypothesis, a warning hypothesis predicts that guiding to nonbees should occur following consistent wax rewards. This was not supported, since we did not detect higher rates of guiding to nonbee animals in areas where honeyguides had been previously rewarded, albeit in a limited sample given the rarity of guiding to nonbee animals (Figure 4C).

Third, we propose as an alternative hypothesis that honeyguides may guide humans to recruit them as mobbing partners toward nonbee animals which they may perceive as a threat (e.g., snakes, predatory birds), much as other birds recruit other species as mobbing partners to help chase away a mutual threat (Dutour et al. 2017). The natural history of these species does not clearly support this idea, since two of the snake species guided to (python and puffadder) are terrestrial and relatively slow‐moving, and small, agile, arboreal birds such as honeyguides are likely not an important part of their diet (Short 1988). In general, species in the honeyguide family (Indicatoridae) are typically cryptic and rarely mob other species (Hockey et al. 2005), and greater honeyguides only make themselves conspicuous when singing or when chattering to elicit cooperation from humans (Fry et al. 1988). Thus, a mobbing function seems unlikely.

Instead, our data were most consistent with a fifth hypothesis (hypothesis v, Table 1): that honeyguides sometimes deliberately guide humans to a location where the honeyguide has previously encountered an animal, and which it has stored in its spatial memory, but then makes a *cognitive spatial recall error* about which locations correspond to a food reward. In this concept, the only difference between the honeyguide's behavior when guiding to bees or nonbees is the erroneous selection of the destination. Spatial recall errors have been widely measured in scatter‐hoarding bird species (Pravosudov and Roth 2013; Sonnenberg et al. 2019), and variation in spatial cognition can be influenced by environmental complexity (Branch et al. 2022; Roth et al. 2012), sleep (Rattenborg et al. 2011), and hippocampal neurogenesis (Leuner et al. 2006). It is also plausible that honeyguides may have genetic variation in their spatial cognitive ability, such as that recently demonstrated in scatter‐hoarding, free‐living mountain chickadees ( *Poecile gambeli* ). Such genetically linked variance makes spatial cognitive ability available for natural selection (Branch et al. 2022; Welklin et al. 2024). A genetic basis for variation in spatial cognitive ability in honeyguides could provide an explanation for why honeyguide spatial error rates appear to be low since there should be strong selection for reliable spatial cognition in honeyguides given the key role of spatial information in honeyguide ecology. Although we currently lack a detailed understanding of honeyguide cognition, we know that honeyguides are ecologically heavily reliant on spatial memory. Both sexes cache food (pieces of high‐quality beeswax: Isack 1987; Lloyd‐Jones et al. 2022) and can directly guide cooperating humans to bees' nests up to 1500 m away (Isack and Reyer 1989), and as brood parasites, females must locate and relocate numerous nests of their hosts (Spottiswoode and Koorevaar 2012).

The honeyguide foraging system for beeswax also has intriguing parallels to systems of caching and food retrieval found in other groups of birds. Corvid species annually find, hide, and retrieve up to 100,000 food items, termed “scatter hoarding” (Clayton et al. 2007; Vander Wall 1990). For example, Western scrub‐jays ( *Aphelocoma californica* ) cache multiple food types year‐round and not only remember the location of food items but also the food type and timing of caching (Correia et al. 2007). In comparison, honeyguides must first locate spatially scattered food sources (bees' nests), store the location of these possible future food sources, and then recall them accurately when the opportunity to guide a honey hunter arises, potentially from a novel direction (Isack and Reyer 1989). Unlike other scatter‐hoarding bird species, which may have more time with which to recall spatial information, honeyguide encounters with humans are largely unpredictable to the bird in both time and space (Isack and Reyer 1989). This means that honeyguides need to make rapid decisions about where to guide a honey hunter based on their current location, making occasional errors a possibility.

Collectively, our field data and historical accounts suggest that honeyguides guide humans to nonbee animals at a low rate which is tolerated by both partners, yet enough to culturally influence Yao honey hunters. In informal discussions with 21 Yao honey hunters, when asked “Why do you reward honeyguides after a honey‐harvest?”, all 21 honey hunters consistently reported that they do so to motivate the honeyguide to (a) continue guiding to bees in the future, and also (b) altruistically guide them to nearby dangerous animals as a “warning.” A low rate of being guided to nonbee animals appears to be tolerated by Yao honey hunters since the cost is similar to that of being guiding to a bees' nest that the human cannot harvest (i.e., no honey benefits for the human, no beeswax benefits for the bird), and they perceive benefits of being warned about a potential threat.

Such human cultural interpretations of even rare experiences of encounters with dangerous animals could disproportionately influence human responses to honeyguide behavior. For example, whether people consistently reward honeyguides or not may be wholly based on a belief in punishment (or lack of altruistic warning) should they fail to do so. Rapid cultural transmission can propagate human knowledge of even rare honeyguide errors, which in turn may promote future cooperation regardless of whether the human perception of that behavior is positive (warning) or negative (punishment).

## 6 Conclusion

This study reviews and (albeit with a small sample) corroborates centuries of Indigenous African cultural knowledge asserting that honeyguides rarely but intentionally guide humans to nonbee animals. Punishment is an appealing and widely repeated explanation, but our evidence suggests that this behavior is unlikely to have evolved for this function, or as a form of altruistic warning to humans, or to recruit mobbing partners. Instead, the available evidence from our limited data best supports the hypothesis that guiding to nonbees arises from cognitive spatial recall errors that carry a low cost to individual honeyguides. Nonetheless, in some honey‐hunting cultures, interpretations of this behavior as punishment or warning may reinforce cultural traditions of rewarding honeyguides with beeswax. Such cultural interpretations may benefit honeyguides at the population level by providing increased overall beeswax rewards (Lloyd‐Jones et al. 2022). Such potential modest benefits may, along with low costs, reduce selection against cognitive errors.

## Author Contributions

**David J. Lloyd‐Jones:** conceptualization (equal), data curation (equal), formal analysis (equal), investigation (equal), methodology (equal), visualization (equal), writing – original draft (equal), writing – review and editing (lead). **Musaji Muamedi:** conceptualization (equal), data curation (equal), writing – review and editing (equal). **Claire N. Spottiswoode:** conceptualization (equal), data curation (supporting), formal analysis (supporting), funding acquisition (lead), investigation (equal), methodology (equal), supervision (lead), writing – original draft (supporting), writing – review and editing (equal).

## Conflicts of Interest

The authors declare no conflicts of interest.

## Data Availability

The code and data for performing the analyses are available on Github (https://github.com/dlloyd‐jones/guiding_to_non_bees/tree/main).

## Appendix 2

### Account of a “False Positive” Report of Being Guided to a Snake

On June 11, 2023 D.J.L., C.N.S, three honey hunters and one other researcher, were guided at 0955 h by a juvenile male greater honeyguide. We followed the honeyguide, who produced stereotypical, vigorous “chatter” calls throughout for 230 m, and upon reaching a large (> 15 m high) *Ficus bussei* tree, lost sight of the bird but could still clearly hear it chattering in the canopy ahead of us. The habitat was riverine scrub with a nearly closed‐canopy at a height of 4–5 m. Most of the group passed the large *Ficus bussei* (continuing to follow the honeyguide ahead) before one of the honey hunters alerted the group to a Mozambique spitting cobra ( *Naja mossambica* ) basking by the *F. bussei*. The snake remained fully visible for c. 10 s before entering a tree cavity, allowing us time to identify it. The honeyguide could be heard giving chatter calls ahead of us at this time, but did not go silent at the location of the snake, nor visibly fly downward toward it. We spent 2 min 50 s at the tree then continued to follow the honeyguide onward for 14 min and 273 m walking distance, whereupon the honeyguide went silent. From that location we could see a large baobab tree ( *Adansonia digitata* ) 79 m ahead, containing two large bee colonies. At the time, none of the honey hunters mentioned that the honeyguide had deliberately guided us to the snake. After finding the bees we questioned the honey hunters for their interpretation of the honeyguide's behavior and they reported: “the honeyguide paused [at the big tree] therefore it deliberately guided us there”. However, considering that the honeyguide did not descend from the canopy, did not go silent at the snake, and subsequently guided us with chattering calls to a tree with bees 273 m away, we infer that the honeyguide did not deliberately guide us to this snake.

## Appendix 3

**TABLE A1:** Acoustic parameters measured using the “warbleR” package in R, to compare “chatter” and “indication” calls emitted by honeyguides. Parameters in **bold** were not used in the principal components analysis (PCA) as they are highly correlated (R^2^ > 0.75) with other variables.

Measure	Description (methods following [23])
duration	Length of signal (in s)
meanfreq	Mean frequency (in kHz). Calculated as the weighted average of the frequency spectrum (i.e., weighted by the amplitude within the supplied band pass)
sd	Standard deviation of frequency (in kHz). Calculated as the weighted standard deviation of the frequency spectrum
freq.median	Median frequency. The frequency at which the frequency spectrum is divided in two frequency intervals of equal energy (in kHz)
freq.Q25	First quartile frequency. The frequency at which the frequency spectrum is divided in two frequency intervals of 25% and 75% energy, respectively (in kHz)
freq.Q75	Third quartile frequency. The frequency at which the frequency spectrum is divided in two frequency intervals of 75% and 25% energy, respectively (in kHz)
freq.IQR	Interquartile frequency range. Frequency range between “freq.Q25” and “freq.Q75”
time. median	Median time. The time at which the time envelope is divided in two time intervals of equal energy (s)
**time.Q25**	First quartile time. The time at which the time envelope is divided in two time intervals of 25% and 75% energy, respectively (s).
**time.Q75**	Third quartile time. The time at which the time envelope is divided in two time intervals of 75% and 25% energy, respectively (s)
**time.IQR**	Interquartile time range. Time range between “time.Q25” and “time.Q75” (s)
skew	Skewness. Asymmetry of the frequency spectrum
kurt	Kurtosis. Peakedness of the frequency spectrum
**sp.ent**	Spectral entropy. Energy distribution of the frequency spectrum. Pure tone ~0; noisy ~1.
time. ent	Time entropy. Energy distribution on the time envelope. ~0 means amplitude concentrated in a specific time point, 1 means amplitude equally distributed across time
entropy	Spectrographic entropy. Product of time and spectral entropy sp.ent * time. ent.
sfm	Spectral flatness. Similar to sp.ent (Pure tone ~0; noisy ~1).
meandom	Average of dominant frequency measured across the spectrogram
mindom	Minimum of dominant frequency measured across the spectrogram
maxdom	Maximum of dominant frequency measure across the spectrogram
dfrange	Range of dominant frequency measured across the spectrogram
modindx	Modulation index. Calculated as the cumulative absolute difference between adjacent measurements of dominant frequencies divided by the dominant frequency range (measured on the spectrogram)
**startdom**	Dominant frequency measurement at the start of the signal (measured on the spectrogram)
**enddom**	Dominant frequency measurement at the end of the signal (measured on the spectrogram)
dfslope	Slope of the change in dominant frequency (measured on the spectrogram) through time
peakf	Peak frequency. Frequency with the highest energy and is measured on the frequency spectrum.
meanpeakf	Mean peak frequency. Frequency with highest energy from the mean frequency spectrum
meanfun	Average of fundamental frequency measured across the acoustic signal
meanfun	Average of fundamental frequency measured across the acoustic signal
minfun	Minimum fundamental frequency measured across the acoustic signal
maxfun	Maximum fundamental frequency measured across the acoustic signal
hn_freq	Mean frequency of the “n” upper harmonics (kHz) (see analyze). Number of harmonics is defined with the argument “nharmonics.” Only measured if harmonicity = TRUE.
hn_width	Mean bandwidth of the “n” upper harmonics (kHz)
harmonics	The amount of energy in upper harmonics, namely the ratio of total spectral power above 1.25 x F0 to the total spectral power below 1.25 x F0 (dB)
HNR	Harmonics‐to‐noise ratio (dB). A measure of the harmonic content

## Appendix 4

Documented reports of honeyguides guiding to animals other than bees, and the adaptive function culturally attributed by humans to this behavior (if provided).

**Table:**

Human cultural group	Location	Quote on guiding to dangerous animals or animals other than bees	Motives attributed	Animal/nonbee object guided to	Reference
Uncertain	Northern Ethiopia	“For there is a little Bird, by those of *Tygra* call'd from the noise which it makes *Pipi*, which, strange to tell will, lead the hunters to the places where the wild beasts lye hid: never leaving their note of *Pipi*, till the hunters follow them, and kill the discover'd prey. Gregory related to me, that as he was walking with one of his acquaintance, an inhabitant of *Tygra*; this bird cry'd *Pipi* over their heads; thereupon, understanding the meaning of it from his friend, he resolv'd to try the truth of the story. The bird conducted them to a shady tree, about the boughs of which, a monstrous huge snake had curl'd her self; at the sight whereof, he and his friend made more hast back again, than they did coming to satisfie their curiosity. And indeed, it is not safe to follow this bird, unless a man be provided with all his hunting instruments: nevertheless, the bird has her own ends in her double diligence too: for she is sure to have her share of the slaughtered carcase whate're it be. Nor is this bird to be found only in *Habessinia*, but also in *Guiny* in the Kingdom of *Quoja*, where they give it the name of *Fonton*, being about the bigness of a Larke, where it is reported to betray not only wild beasts, but also serpents and bees.”	Benefits from eating the carcass	Snake, wild beasts	Ludolphus 1682
Uncertain	Congo Basin	“Nor is there less to be admired in another bird in these parts, and particularly the kingdom of Matamba, which as travellers are on their way, harmoniously sings, *Vuichi, Vuichi*, which in the language of the Blacks signifies, *honey, honey*; and flopping from one place to another, rest upon the tree where the honey is, that the passengers may take it, and the bird feed on what remains. But it sometimes falls out, that following the cry of the bird, the passenger falls into the clutches of some lion that lies hid, and so meets his death instead of honey; therefore when the bird cries, if he sees not the honey, they are aware of the hidden lion, and fly in time.”	Punishment	Lion	da Sorrento 1744
Khoe‐Sān	Western Cape, South Africa	“The inhabitants in general accuse the same bird of sometimes conducting its followers where wild beasts and venomous serpents have their places of abode: this however I never had an opportunity of ascertaining myself; but am apt to believe such cases to be accidental, when dangerous animals happen to be in the neighbourhood of a bees‐nest.”	Incidental	Wild beasts and venomous snakes	Sparrman 1777
Khoe‐Sān	KwaZulu‐Natal, North West Province, South Africa	“A number of travelers have described the characteristics of the honey‐guide and maintain that it is spared by the hunter because of its usefulness to man. Somebody has said, and Buffon has repeated it, that there is danger in following this bird as it will often lead the hunter into the clutches of wild animals, such as lions and leopards. At first, when I has studied the honey‐guide only in the Amazoulou [Zulu] country, I was tempted to reject this theory as a fable for in those parts the bird appeared to have no instinct other than the one to which it owes its name; it led us to the hives and only to the hives; and so we had to reason to mistrust it. But later, beyond Malalis‐Berg [Magaliesberg], I watched my men set out on numerous fruitless searches in answer to the bird's call; after ten or fifteen vain attempts, I heard them cursing the *om‐schlanvo*; one of them had twice been led to a rhinoceros skeleton, another to a dead buffalo and a third to the bones of a *canna*. Henning himself, who loved honey, had been deceived on four occasions; each time he had been led to a skeleton and twice the hyaenas had fled at his approach. One must not conclude from these facts that the cuckoo [honeyguide] has an understanding with the great carnivores to bring them men to eat, by rather that, feeding on insects, this fragile bird has the need of the assistance of man to move the bones beneath which swarm beetles and other insects, and which the cuckoo [honeyguide] is unable to reach while they are hidden. It is only just and fair that, when he cannot himself obtain the food which is necessary for his existence, this bird will ask assistance of man who he has already helped to procure the sweets contained by nature.”	To gain access to insects upon which to feed	Lions, leopards, rhino skeleton, dead buffalo	Delegorgue 1847
Uncertain	Limpopo, South Africa	“Interesting as the honey‐bird is, and though sweet be the stores to which it leads, I have often had cause to wish it far enough, as, when following the warm ‘spoor’ or track of elephants, I have often seen my [trackers] at moments of the utmost importance, resign the spoor of the beasts to attend to the summons of the bird. Sometimes, however, they are ‘sold’, it being a well‐known fact, both among the Hottentots and tribes of the interior, that they often lead the unwary pursuer to danger, sometimes guiding him to the mid‐day retreat of a grizzly lion, or bringing him suddenly upon the den of the crouching panther. I remember on one occasion, about three years later, when weary with warring against the mighty elephants and hippopotami which roam the vast forests and sport in the floods of the fair Limpopo, having mounted a pair of unwonted shot‐barrels, I sought recreation in the humbler pursuit of quail‐shooting. While thus employed, my attention was suddenly invited by a garrulous honeybird, which pertinaciously adhered to me for a considerable time, heedless of the reports made by my gun. Having bagged as many quails and partridges as I cared about shooting, I whistled lustily to the honeybird, and gave him chase; after following him to a distance of upward of a mile, through the open glades adjoining the Limpopo, he led me to an unusually vast crocodile, who was lying with his entire body concealed, nothing but his horrid head being visible above the surface of the water, his eyes anxiously watching the movements of eight or ten large bull buffaloes, which, in seeking to quench their thirst in the waters of the river, were crackling through the dry reeds as they cautiously waded in the deep mud that a recent flood had deposited along the edge. Fortunately for the buffaloes, the depth of the mud prevented their reaching the stream, and thus the scaly monster of the river was disappointed of his prey.”	Unattributed	Lion, “panther” [leopard] Crocodile	Cumming 1856
Uncertain	Northern modern day Zambia	“DECEMBER 2, 1855. We remained near a small hill, called Maundo, where we began to be frequently invited by the honey‐guide (‘Cuculus indicator’). Wishing to ascertain the truth of the native assertion that this bird is a deceiver, and by its call sometimes leads to a wild beast and not to honey, I inquired if any of my men had ever been led by this friendly little bird to any thing else than what its name implies. Only one of the 114 could say he had been led to an elephant instead of a hive, like myself with the black rhinoceros mentioned before. I am quite convinced that the majority of people who commit themselves to its guidance are led to honey, and to it alone.”	Unattributed	Elephant	Livingstone 1858
Wakamba	East Africa	“East African natives, so far as I am aware, have no stories of guides wreaking their vengeance on the man who omits to leave some honey for them by leading him up to dangerous animals, as have the natives of South Africa… I have been told of cases in which men have been led to snakes and dangerous beasts, but these must be discounted by the obvious chances of the bush. When men take a ‘beeline’ through the bush, whether following a honey‐guide or not, there is always the chance of happening on dangerous animals or the reverse. My own boys have followed for honey hundreds, perhaps one thousand times; once they returned to say that the bird led them up to buffalo. I have very often followed myself, and was once thus brought to a wounded lion. I was as firmly persuaded then that the thing was intentional as I am now that it was not.”	Incidental	Snakes, dangerous animals, buffalo, wounded lion	Percival 1924
Uncertain	East Africa	“In East Africa… there is a chance of being led by the honey‐guide to a lion or leopard. This would seem to be mere accident, but Blayney Percival [12; A Game Ranger's Notebook] vouches for one case in his own experience.”	Unattributed	Lion, Leopard	Chapin 1939
Uncertain	Northern Zimbabwe	“Natives often make use of the fact that very occasionally a honeyguide may appear to lead to a dangerous animal instead of a bees' nest, to impute a revenge motive to the bird. This has given rise to a variety of proverbs, such as: If you do not leave anything for the guide, it will lead you to a dangerous animal the next time. If you do not leave anything for the guide, it will not lead you at all in the future. If you do not leave anything for the guide, it will come to you and chatter when you are stalking game, and thus reveal our presence to the intended quarry. “Still another bit of evidence as to the nature of the performance is the fact that on occasions the bird will lead not to a bees' nest but to a dead animal or to a live snake, leopard, rhinoceros, etc. I doubt that these are in any sense the “purposive” goals behind the species habit of guiding, and that in most cases they may be dismissed quite easily as distracting incidents on the guiding trip that cause the guide and, more certainly, the follower to “forget” the original “goal.” “Major Haydock, in Northern Rhodesia, was told by his native collectors that when a honey‐guide led them to a leopard or a lion it was always a sleeping beast, and that the bird seemed to mistake the flies hovering about it for bees (this is the natives' own, unaided conclusion). It seems very probable that the real explanation is that the flies hovering about may have a similar effect on the honey‐guide as would a flight of bees. In some of these instances the observers made particular search to see if there might have been a bees' nest near the animal; if so, the animal's presence could not be looked upon as more than a coincidence, but in a number of cases there was no sign of a hive. It might be mentioned, parenthetically, that there are some who think that the actions of the bird may be slightly different when coming to a large animal than when arriving at a bees' nest.”	Revenge motive (punishment), distraction while guiding, punishment	Live snake, leopard, rhinocerous	Friedmann 1955 1955
Uncertain	Eastern Cape Province, South Africa	“The natives on the farm, when asked, repeated the story of birds guiding people to snakes but in the absence of actual instances this seems illusory. In addition they referred to people being led to dead animals, but this I believe to be apocryphal…”	Unattributed	Snakes, dead animals	Skead 1951
Uncertain	Southern Tanzania	“By constantly twittering and nagging till you follow, a honey guide will lead you to a beehive. It wants you to get rid of the bees and open the hive, being interested in the grubs and honey. It is usual to leave some honey for the bird, and there is a belief among Africans that if this is not done it will lead the next man it sees to a lion or snake. There is evidence of the truth of this behaviour ‐ though not the bird's motives ‐ because I have on three occasions been led by it to things other than honey, and this was one of them. It would fly around us twittering insistently, then go over to the man‐eater: it was fairly open country and we could follow the passage of our quarry by the birds suddenly getting up from bushes as it passed under. We came upon several places where the lion had tried to lie down and rest, but had got up and moved on because of the racket this infernal bird was making. This performance went on quite a long time, till the lion suddenly bolted away, probably in an attempt to shake off the pest.”	Punishment	Lion or Snake	Ionides 1965
Senga	Chikwa, Northern Zambia	“Because of this Nsulu [greater honeyguide] commands a great respect among the tribes and much of their folklore and legends centre round the birds, as around any creature which touches the economy of their lives. There are certain rites, too, which must be followed when honey is taken from the hive. It is important for instance to leave a fair portion of the comb for the honey‐guide in payment for services rendered. This act must be performed with deference and the appropriate obeisance's. And woe betide anyone who neglects this little courtesy. There is no doubt in the minds of these people that such an offender against the properties will be adequately punished; the next time he follows Nsulu he will certainly be led into the clutches of a waiting lion, or toward any of the other fates that await evil‐doers.”	Punishment	Lion	Carr 1969
Boran	Northern Kenya	“A common belief that the Boran share with several other communities in Africa is that the bird often guides man to animals such as lions, buffalos, elephants, rhinos, large poisonous snakes or to enemies or murderers from a hostile tribe. They are also said to lead someone to old weapons (bows, arrows, spears) and hidden trophies such as elephant tusks and rhino horns. It is believed that the bird resorts to this abnormal result of man's failure to spare some food for it after the bird had helped him discover a colony. Some people claim that as soon as a bird arrives and start guiding, they can tell whether it has such a motive or not. This, they say, can be established by observing the guiding behavior of the bird during the trip. Others claim that there are two types of honeyguides: the good ones which guide to bee colonies, and the rogue ones who specialize in guiding to dangerous destinations.”	Punishment	Lions, buffalos, elephants, rhinos, large poisonous snakes, old weapons (bows, arrows, spears), hidden trophies such as elephant tusks and rhino horns	Isack 1987
Wataa	Tsavo, southern Kenya	“It was a woman's task to hunt for the nests of wild bees found in trees or in the ground, sometimes with the help of the greater honeyguide ( *Indicator indicator* ) although this bird may bring you to a rhino or a snake instead.”	Unattributed	Rhino, Snake	Ville 1995
Boran	Northern Kenya	“Does the bird guide man to dangerous animals? Contrary to the traditional belief that the honeyguide often leads people to dangerous objects, I have never witnessed such an event, except on coincidental situations. However this belief is so widespread across the whole continent of Africa that a study of it may be warranted.”	Incidental	Dangerous (to human) animals	Isack 1999
Uncertain	Kenya	“There is a considerable literature (Friedmann 1955) on Greater Honeyguides ‘guiding’ humans to lions, or poisonous snakes, or the presence of elephants. Persons with sufficient field experience will be aware that such animals are of much greater consequence to humans than to the guiding Greater Honeyguide in a tree above. We have been ‘guided’ to an abrupt precipice and to a bull elephant by Greater Honeyguides. In these cases there were bee‐hives below the cliff (in a valley) and beyond the elephant. Concern for the welfare of the guided person is beyond any reasonable expectation of a honeyguide, yet it is easy to appreciate how an untrained or unobservant individual could interpret such ‘evil’ guiding wrongly.”	Incidental en route to bees	Lions, snakes, elephants	Short and Horne 2001
Batonga	Southern Zambia	“ ‘One honeyguide’, Lazaro [Hamusikili] said, ‘showed me four hives; one hive then another then another; and sometimes, because it is interested, it showed us other things as well, a snake or dead animal in the bush’.”	Out of interest	Snake, dead animal	Dee 2013
Fulani, Mbum, Baya	Central Cameroon	“We tried to locate the bird and climbed down a steep slope toward a small riverbed, but the vegetation was too dense to even see it. After a few minutes, as we were unable to follow the bird, the chattering slowly moved further away and eventually vanished in the distance. When the bird was gone, our guide immediately pointed to a hole in the ground—a snake's hole he said—and explained that the honeyguide probably wanted to warn us of a snake (See Figure 2). In that moment, it sounded to me like an excuse for the ethnographers' benefit. However, the bird is frequently and widely reported to indicate the presence of dangerous animals. Friedmann dismisses such reports as ‘native legends’ or as coincidental (1955: 57). Our interlocutors told us that when this happens, the bird wants to warn them. For hunters of game this may have negative effects as the honeyguides' calls may also warn and chase away their prey. Unfortunately, the phenomenon has not been followed up and further research is necessary…”	Warning	Dangerous (to human) animals, Snake	Gruber 2018
Maasai	Northern Tanzania	“For example, Maasai respondents reported using simple words (“enjarwatai”, meaning “sheep”) mixed with trill‐grunts when attracting honeyguides. But when a honeyguide starts to guide them, they then switch to more complex messages interspersed among trill‐grunts (transcribed here as “Prrr, Hii”) as guiding calls… which approximately means “Please my dear sheep, please show me the location of the honey, I can harvest it while I am kneeling or standing from the tree or from the termite mound, but please don't lead me to a dangerous animal.”	Unattributed	Dangerous (to human) animals	Laltaika 2021
Awer	Northern Kenya	“All interviewees also reported that they have sometimes been guided to animals other than bees, namely snakes (*n* = 4 interviewees), lions (*n* = 5 interviewees) and other animals (*n* = 6 interviewees). These events were not interpreted in any particular way, and one interviewee explicitly attributed this to chance.”	Unattributed, Incidental (when attributed)	Snakes, lions, other animals	van der Wal et al. 2022
